# Prevalence and factors associated with dyslipidemia among adolescents in Saudi Arabia

**DOI:** 10.1038/s41598-022-21262-9

**Published:** 2022-10-07

**Authors:** Shadan AlMuhaidib, Fadia AlBuhairan, Waleed Tamimi, Mohammed AlDubayee, Aqeel AlAqeel, Amir Babiker, Haifa AlFaraidi, Fahad AlJuraibah, Motasim Badri, Ibrahim Al Alwan

**Affiliations:** 1 Department of Epidemiology and Biostatistics, College of Public Health and Health Informatics, King Saud bin Abdulaziz University for Health Sciences, Riyadh, Saudi Arabia; 2 Department of Central Military Laboratory and Blood Bank, Prince Sultan Military Medical City, Riyadh, Saudi Arabia; 3 Health Sector Transformation Program, Riyadh, Saudi Arabia; 4 College of Medicine, Alfaisal University, Riyadh, Saudi Arabia; 5 Department of Pathology and Laboratory Medicine, King Abdulaziz Medical City, Riyadh, Saudi Arabia; 6 College of Medicine, King Saud Bin Abdulaziz University for Health Sciences, Riyadh, Saudi Arabia; 7 Department of Pediatrics, King Abdullah Specialized Children Hospital, Ministry of the National Guard Health Affairs, Riyadh, Saudi Arabia; 8 King Abdullah International Medical Research Centre, Riyadh, Saudi Arabia; 9 Department of Pediatrics, College of Medicine, Qassim University, Buraydah, Qassim Saudi Arabia

**Keywords:** Dyslipidaemias, Public health

## Abstract

Dyslipidemia is a major risk factor for atherosclerosis. Screening for dyslipidemia at an early age is essential to prevent and control its consequences. This study aimed to determine prevalence of dyslipidemia and its correlates among adolescents in Saudi Arabia. Data of 5854 adolescents aged 10–19 years from all 13 regions of Saudi Arabia were obtained from the Jeeluna study; a national cross-sectional, multistage stratified cluster sample survey. Dyslipidemia was defined based on the National Heart Lung and Blood Institute and National Cholesterol Education Program guidelines for adolescents. We found that a quarter of Saudi adolescents have dyslipidemia (males: 33.3%, females: 17.9%). Significant variation was observed by region (*p* < 0.001). Prevalence of abnormal Total Cholesterol was 6.7%, LDL-C 7.1%, HDL-C 12.8%, Non-HDL-C 8.3%, and Triglycerides 9.6%. Factors independently associated with dyslipidemia were male gender (OR = 2.19, 95% CI 1.78–2.70, *p* < 0.001), BMI (underweight OR = 0.80, 95% CI 0.69–0.94, overweight OR = 1.76, 95% CI 1.50–2.06, obese OR = 2.80, 95% CI 2.34–3.34, *p* < 0.001, vs. normal) and serum ferritin (high OR = 7.02, 95% CI 1.49–34.79, low OR = 0.82, 95% CI 0.67–1.01, *p* = 0.04 vs. normal) and ≥ 1 daily intake of carbonated beverage (OR = 1.10, 95% CI 1.00–1.20, *p* = 0.03 vs. no or not daily intake). Public health interventions for improving lipid profile of adolescents are urgently needed.

## Introduction

Dyslipidemia is defined as an elevation in total cholesterol (TC), low-density lipoprotein (LDL-C), triglycerides (TG), non-high-density lipoprotein (Non-HDL-C) or decreased high-density lipoprotein cholesterol (HDL-C)^1,2^. Dyslipidemia can be categorized into primary dyslipidemia, which is due to genetic predisposition, and secondary dyslipidemia, which is multifactorial and is typically related to an underlying medical condition^1,2^. Multifactorial dyslipidemia is usually associated with behavioral factors, dietary habits, and environmental factors, and it could be with or without genetic inheritance^2^. Dyslipidemia is a well-known risk for atherosclerosis, which is a major risk factor for cardiovascular disease and premature mortality^3^. A report published by the World Health Organization (WHO) found that 4.5% of the global death rate and 2% of disability-adjusted life years (DALYs) in both genders aged 18 years and over are due to the high level of cholesterol^4^.

Most guidelines recommend universal screening of the lipid profile to be performed between 9–11 years of age and again at 17–21 years on non-fasting samples, in order to detect lipid abnormalities at an early stage. If a lipid abnormality is detected, confirmation is thereafter done with a fasting sample^1,5^. Moreover, results of the National Health and Nutrition Examination Surveys (1999–2008) for children and adolescents showed minor variations between the use of fasting and non-fasting serum samples in lipid assessments which are expected to be clinically insignificant^6^. In 2018, the global prevalence estimated from the WHO regions for high total cholesterol was 39% in adults (both genders)^7^. Whereas prevalence of elevated total cholesterol among the Saudi population aged 15–64 years in a cross-sectional study done in 2005 was 20%, and prevalence of dyslipidemia, in general, ranged between 20 and 40%^8^.

The global burden of dyslipidemias has risen in the last 30 years^9^. The current global public health goal is to decrease 25% of the premature death rate due to chronic and non-communicable diseases by 2025 and assessing dyslipidemia and early treatment might support reaching the desired goal^10^.

Jeeluna’s study found that 30% of all adolescents in Saudi Arabia are overweight (14.1%) or obese (15.9%), and 44.3% were physically inactive^11^. Additionally, previous Saudi studies among adolescents reported a high prevalence of poor dietary habits and sedentary lifestyles, which is expected to have a negative impact on adolescents’ health and well-being^11–14^.

Since it is well-known that lipid abnormalities are related to these factors, and most of the non-communicable disorders in adulthood life start at adolescence age, and little is known about dyslipidemia among Saudi adolescents, the assessment is critical. Moreover, published studies have several limitations, including limited sample sizes within specific communities/regions and addressing aspects of the lipid profile parameters while using adult ranges in some instances. None of these studies can therefore be generalized to the overall adolescent population^15–17^. Therefore, we conducted this study to determine prevalence of dyslipidemia and its associated factors among Saudi adolescents by analyzing data from a large population-based cross-sectional study; the Jeeluna (our generation) study. We believe our results will provide valuable representative information regarding the prevalence of dyslipidemia among adolescents in Saudi Arabia and facilitate understanding the underlying factors in order to address them better.

## Methods

The Jeeluna study is a national population-based study conducted in 2011–2012 on adolescents aged 10–19 years attending intermediate and secondary schools throughout all 13 regions of the Kingdom of Saudi Arabia. The overall study aimed at identifying the health status and needs of adolescents in the country. It addressed health holistically, with the biopsychosocial approach to health.

### Study design

The details of the Jeeluna study and its methods have been previously published^11,13^. In brief, it is a cross-sectional study, with participants selected using a multi-stage, stratified, cluster, random sampling technique based on student population per region, district, gender, and school level. A comprehensive list of intermediate and secondary schools was obtained from the Ministry of Health and served as the overall sampling frame. Sample size allocation was proportionate to region student population. Schools, and classes within selected schools, were randomly selected using computer-based randomized sampling. An information letter was sent to students and parents, and their informed consent were obtained. Students were given the option to opt-out of blood sampling. The Jeeluna study protocol was approved by the Institutional Review Board and the Ethics Committees at the King Abdullah International Medical Research Center (KAIMRC) (Protocol RC08-092). All procedures performed in this study were in accordance with the ethical principles and professional conduct standards of the Helsinki declaration.

### Data collection

Data collection included a self-administered questionnaire, anthropometric measurements and blood sampling for the laboratory diagnosis.

#### Demographics and survey measures

*Self-administered questionnaire* The questionnaire included several domains; however, only relevant domains to dyslipidemia were considered in the current study. These include: socio-demographic, fasting state, history of chronic disorders or diabetes mellites, nutrition/dietary behaviors, activities, and tobacco and substance use.

#### Clinical anthropometrics

Height was rounded to the proximate 0.5 cm and weight measured by an electronic scale (OmronSC100 digital scale, USA) and rounded to the nearest 0.1 kg. The calculated body mass index (BMI) was plotted on the Center for Disease Control and Prevention (CDC) BMI charts^18^. Based on the norm for age and gender, BMI percentiles were expressed as underweight, normal weight, overweight, or obese if < 5th, 5th to < 85th, 85th to < 95th, or ≥ 95th, respectively.

#### Blood pressure measurements

Blood pressure (BP) was measured twice a few minutes apart using a digital BP monitor (Omron M2, Netherlands) on the right upper arm, and the average was recorded.

#### Laboratory investigations

Two blood samples were obtained from each student enrolled in the study. The samples were packed and transported to the hospital laboratory upon collection at a cold temperature. Serum gel separator tubes were used to collect the blood for calcium, ferritin, alkaline phosphatase and phosphate tests. The EDTA blood tubes were used to collect blood for complete blood cells (CBC) analysis. The EDTA tubes were transferred immediately to the laboratory to be analyzed within 24 h. Serum gel separator samples were kept for one hours to be clotted before it centrifuged for 10–15 min at 3000 rounds/minutes to obtain the serum. Chemistry tests were performed using automated chemistry analyzer (Architect c16000; Abbott, USA) and an immunoassay analyzer (Architect i2000; Abbott, USA) for hormones. EDTA tubes were analyzed immediately upon arrival to the lab by an automated hematology cell counter analyzer (Abbott, USA).

For the purpose of this study, we included participants from the Jeeluna study who were tested for the lipid profile panel. Those who did not consent to draw blood samples or had at least one missing value of the lipid profile panel or aged < 10 or > 19 years old were excluded. Definition of dyslipidemia is based on the National Heart, Lung, and Blood Institute (NHLBI) recommendation for testing lipid profile panel, which stipulates that a non-fasting blood sample should be used for the first time and if any abnormalities are observed, a selective fasting sample should be requested^3^. Therefore, fasting, non-fasting, and unknown state of fasting samples were included in the analysis. The lipid profile panel results were categorized based on the universal screening cut-offs for adolescents from both the NHLBI and NCEP guidelines^5,19^. The participant’s lipid profile values were measured in mmol/L; therefore, the universal cut-off thresholds were converted from mg/dL into mmol/L. The total cholesterol, HDL-C, Non-HDL-C and LDL-C were divided by 38.67, and triglyceride was divided by 88.57^20^. The cut-off points of lipid profile in mmol/L were classified into three categories, which are acceptable, borderline, or at high-risk respectively: total cholesterol (TC): (< 4.40, 4.40–5.16, and ≥ 5.17); low-density lipoprotein cholesterol (LDL-C): (< 2.84, 2.84–3.39, and ≥ 3.4); high-density lipoprotein cholesterol (HDL-C): (> 1.17, 1.01–1.17, and ≤ 1); non-high-density lipoprotein (Non-HDL-C): (< 3.11, 3.11–3.74, and ≥ 3.75); and triglycerides: (< 1.02, 1.02–1.45, and ≥ 1.46).

Participants were categorized as dyslipidemic (at least one of the lipid parameters in the high-risk category) or non-dyslipidemic (all the lipid parameters are either in accepted and/or borderline categories). Blood pressure was considered elevated if: ≥ 90th percentile to < 95th percentile or 120/80 mm Hg to < 95th percentile (whichever is lower)^21^. All laboratory tests results were categorized based on the manufacturer reference range and were classified as normal, abnormally low, or abnormally high, considering variations in age and gender.

### Statistical analysis

Continuous variables were initially tested for normality using the Shapiro–Wilks test and were accordingly expressed as means (SD) or medians (IQR) and compared using the *t*-test or non-parametric Mann–Whitney test. Categorical variables were expressed as frequencies (%) and compared using the *χ*^2^ test. Data other than lipid variables were considered for imputation if their missing data were 20% or less. However, most of the included factors had 0–5.6% missing data, with the exception of blood pressure measurements which had approximately 15% missing data. We conducted a binary logistic regression analysis to identify factors associated with dyslipidemia using the Complex Sample module of the IBM SPSS software (Version 28, IBM Corp, NY, USA) to adjust for complex sampling design. Factors found significant in the univariate analysis were included in the final multivariate model. All tests were two-sided and a *p* value ≤ 0.05 was considered statistically significant.

## Results

### Demographic and clinical characteristic of the study participants

The study included 5854 participants with 2973 (50.8%) females. Mean (SD) age was 15.84 (1.8), and median (IQR) 16 (3). Seventy-four percent of participants were late adolescence (15–19 years) and the rest were early adolescent age (10–14 years). Participants who suffered from chronic illnesses were 8%. Additional characteristics are presented in Table 1. Moreover, there were differences between males' and females’ BMI results; 13.5% and 19.6% of males compared to 15.6% and 11.5% of females were overweight and obese, respectively.

**Table 1:** General characteristics of the study participants.

Characteristics	Total N (%)
**Age (years)**	
Median (IQR)	16 (3)
Mean (SD)	15.84 (1.8)
Early adolescence (10–14 years)	1523 (26)
Late adolescences (15–19 years)	4331 (74)
**Gender**	
Female	2973 (50.8)
Male	2881 (49.2)
**BMI**	
Underweight	820 (14)
Normal	3273 (55.9)
Overweight	854 (14.6)
Obese	907 (15.5)
**Fasting (> 8 h)**	
Yes	2787 (47.6)
No	1156 (19.7)
Unknown	1911 (32.6)
**Chronic illness**	
Yes	470 (8)
No	5384 (92)
**Diabetes mellitus**	
Yes	33 (0.6)
No	5821 (99.4)
**Region**	
Riyadh	1596 (27.3)
Makkah	1360 (23.4)
Eastern Province	646 (11)
Madinah	453 (7.7)
Asir	401 (6.9)
Qassim	293 (5)
Hail	226 (3.9)
Aljouf	220 (3.8)
Tabuk	151 (2.6)
Northern Borders	148 (2.5)
Albahah	128 (2.2)
Najran	119 (2)
Jizan	104 (1.8)

*%* percentage, *BMI*, body mass index, *hr* hour, *N* number of participants.

### Prevalence of dyslipidemia

**Figure 1:**
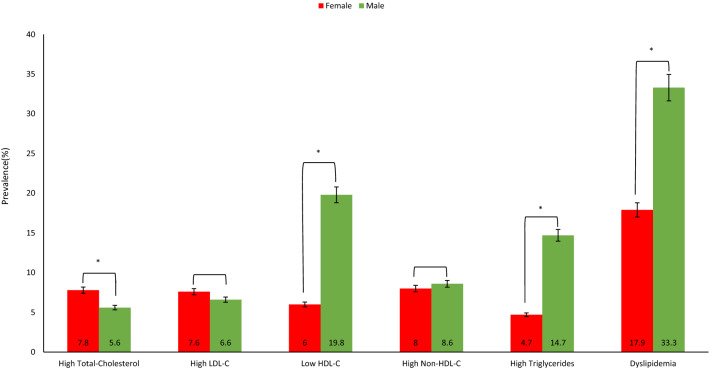
Prevalence of abnormal lipid parameters by to gender. Abbreviations: HDL-C, high-density lipoprotein cholesterol; LDL-C, low-density lipoprotein cholesterol. (*): significant *p* value.

The overall prevalence of dyslipidemia among adolescents was (n = 1493; 25.5%) (at least one abnormal lipid level). Approximately one-third of participants (n = 2116; 36.1%) had normal results of all their lipid profile panel parameters, compared to 3738 (63.9%) participants who had at least one parameter result classified as borderline and/or high-risk. The evaluation for accepted, borderline, and high-risk prevalence for each of the lipid profile panel parameters are shown in Table 2. The Venn diagram in Fig. 1 shows dyslipidemia prevalence for single and multiple lipid abnormalities.

**Table 2:** Evaluation of the lipid profile panel tests.

Parameter	Accepted N (%)	Borderline N (%)	High-risk N (%)
Total-cholesterol	4042 (69)	1420 (24.3)	392 (6.7)
LDL-C	4398 (75.1)	1039 (17.7)	417 (7.1)
HDL-C	3818 (65.2)	1288 (22)	748 (12.8)
Non-HDL-C	4144 (70.8)	1224 (20.9)	486 (8.3)
Triglycerides	4303 (73.5)	987 (16.9)	564 (9.6)

*HDL-C* high-density lipoprotein cholesterol, *LDL-C* low-density lipoprotein cholesterol, *Non-HDL-C* non-high-density lipoprotein.

### Prevalence of dyslipidemia across geographical regions in Saudi Arabia

**Figure 2:**
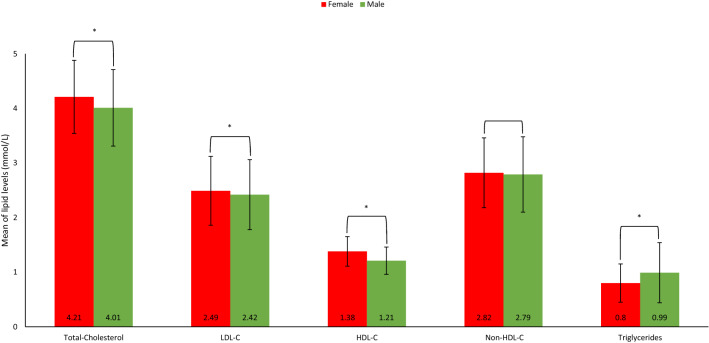
Mean and SD of lipid profile parameters according to gender. Abbreviations: HDL-C, high-density lipoprotein cholesterol; LDL-C, low-density lipoprotein cholesterol; P, *P* value; SD, Standard deviation. (*): significant *p* value.

**Figure 3:**
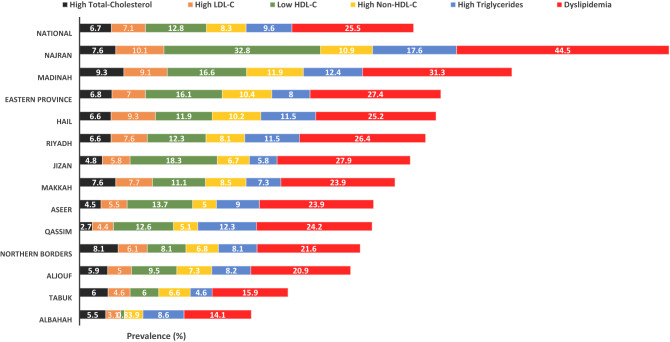
Lipid parameters; nationally and by geographical region. Numbers represent prevalence (%) of each parameter among participants in each region. Difference across geographical regions was statistically significant for dyslipidemia (*p* < 0.001), high triglycerides (*p* < 0.001), high Non-HDL-C (*p* = 0.005) and low HDL-C (*p* < 0.001), but not for high LDL-C (*p* = 0.109) or high total-cholesterol (*p* = 0.089).

The map in Fig. 2 shows prevalence rates of dyslipidemia among adolescents in all the 13 regions of Saudi Arabia. Adolescents in Najran had the highest prevalence of dyslipidemia with a prevalence rate of 44.5% while the lowest was in Albahah regions (14.1%). Differences across geographical regions were statistically significant for dyslipidemia (*p* < 0.001), high triglycerides (*p* < 0.001), high Non-HDL-C (*p* = 0.01) and low HDL-C (*p* < 0.001), but not for high LDL-C (*p* = 0.11) or high total-cholesterol (*p* = 0.09), Fig. 3.

### Sex-specific prevalence of dyslipidemia

**Figure 4:**
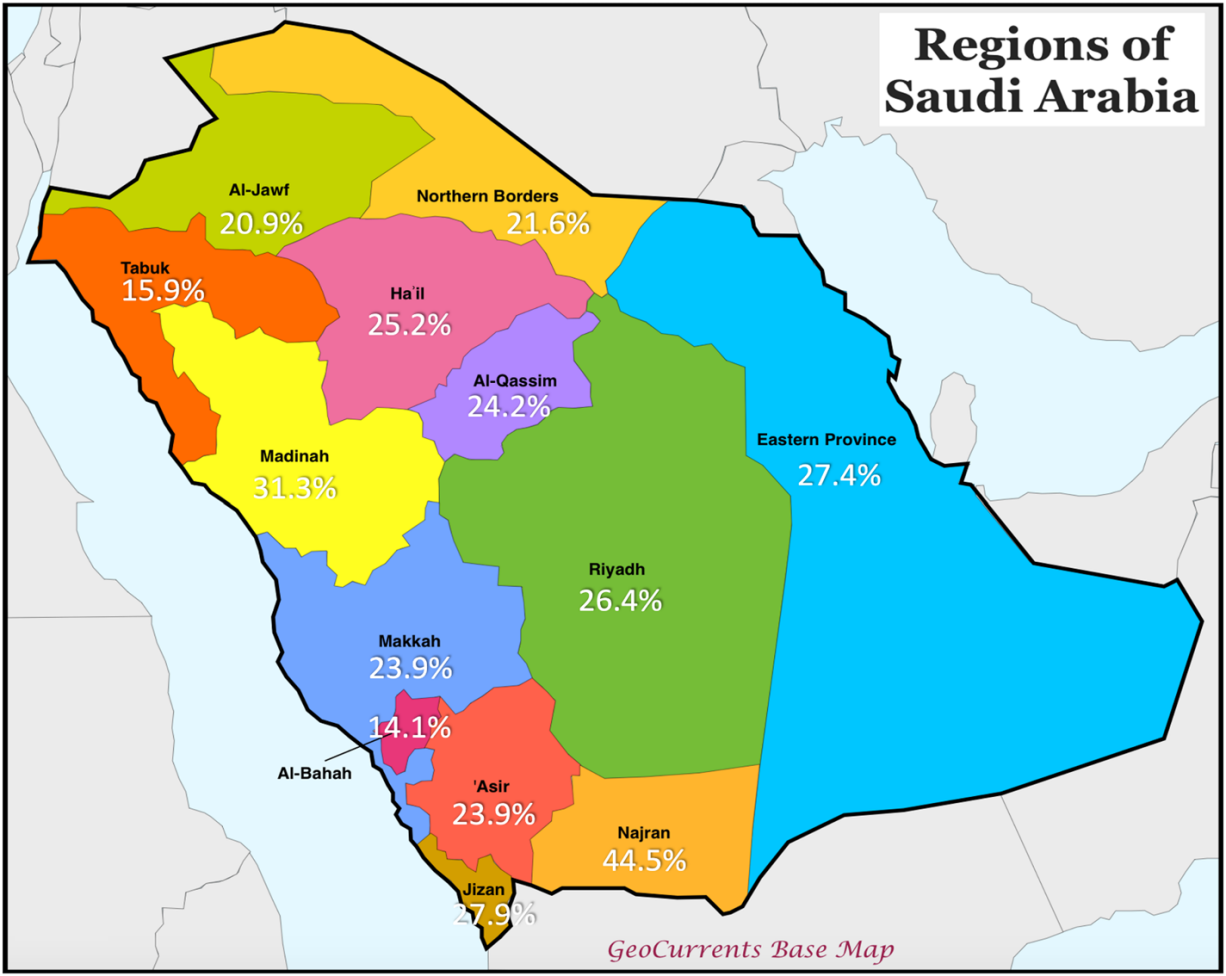
Prevalence rates of dyslipidemia in the 13 regions of Saudi Arabia. Modified from GeoCurrents Base Map. https://www.geocurrents.info/customizable-base-maps.

The sex-based differences for the prevalence of abnormal lipid parameters are shown in Fig. 4. Prevalence of high total-cholesterol was statistically significantly higher among female, in contrast, males were about 3 times higher in prevalence of low HDL-C and high triglycerides as in females, and the total dyslipidemia was around the double in males as in females. However, there was no statistical differences in prevalence of high LDL-C and Non-HDL-C according to sex.

### Sex-specific mean of lipid levels

**Figure 5:**
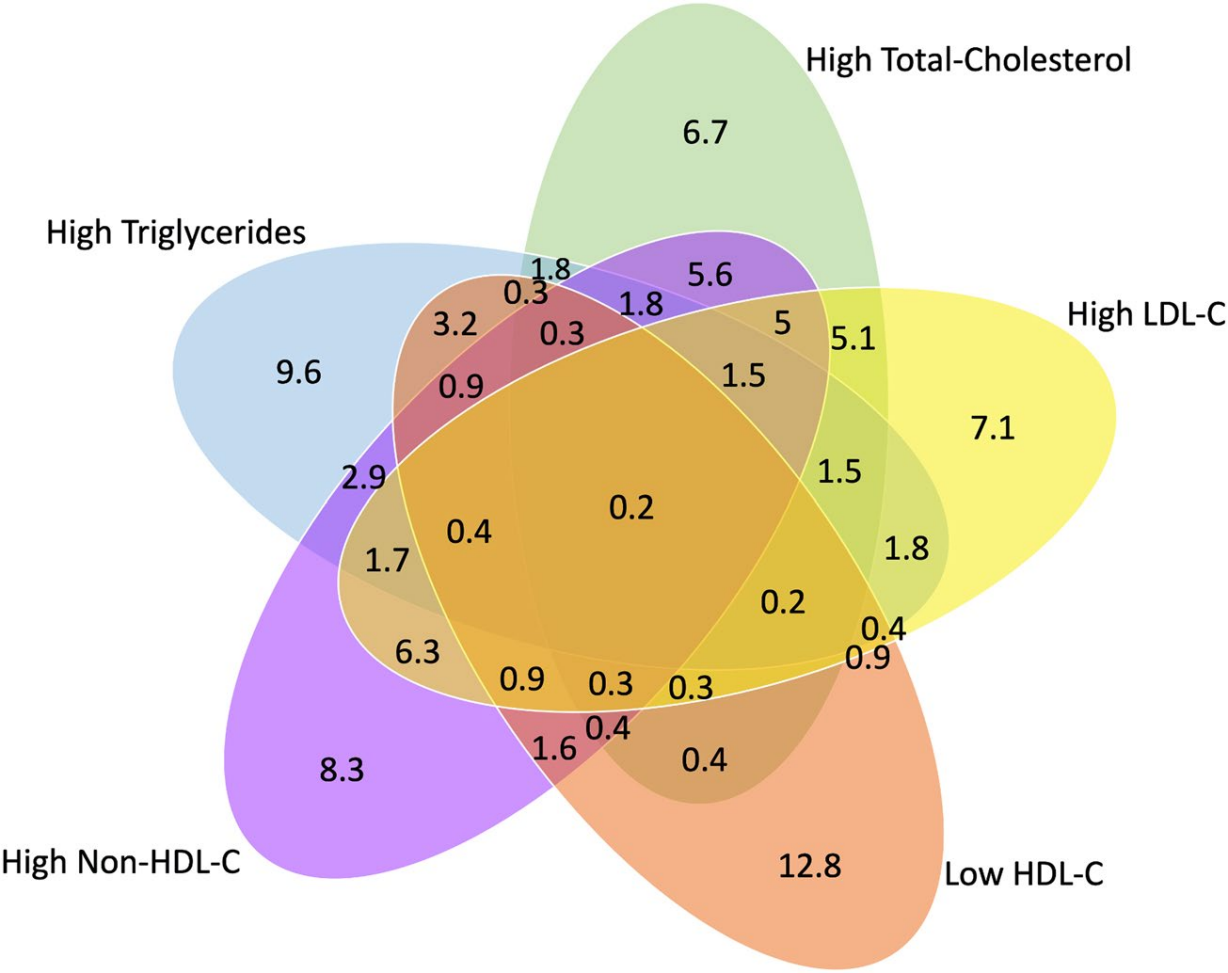
Venn diagram comparing the prevalence of single and multiple lipid abnormalities in adolescents in Saudi Arabia. The values demonstrate the prevalence (%) of abnormally elevated levels of Total-Cholesterol, LDL-C, Non-HDL-C, and Triglycerides, and lower levels of HDL-C. The overlap of sets represents the rates of adolescents meeting more than one abnormality (PowerPoint version 16.63.1).

Lipid parameters (mmol/L) of males and females are shown in Fig. 5. The means of total cholesterol and LDL-C were statistically significantly higher in females compared with males. Whereas lower means of HDL-C and higher triglycerides were observed among males. Moreover, there was no statistical differences based on sex for the means of Non-HDL-C.

### Prevalence of dyslipidemia by patients’ characteristics

Characteristics of dyslipidemic and non-dyslipidemic groups are compared and presented in Table 3.

**Table 3:** Characteristics of dyslipidemic and non-dyslipidemic adolescents.

Characteristics	Dyslipidemic N (%)	Non-dyslipidemic N (%)	*P*
**Age (years)**			
Median (IQR)	16 (2)	16 (3)	**0.01**
Mean (SD)	15.96 (1.82)	15.79 (1.79)	
Early adolescence (10–14 years)	355 (23.8)	1168 (26.8)	**0**.**02**
Late adolescences (15–19 years)	1138 (76.2)	3193 (73.2)	
**Gender**			** < 0**.**001**
Female	533 (35.7)	2440 (56)	
Male	960 (64.3)	1921 (44)	
**Chronic illness**			0.07
No	1390 (93.1)	3994 (91.6)	
Yes	103 (6.9)	367 (8.4)	
**Diabetes mellitus**			0.07
No	1480 (99.1)	4341 (99.5)	
Yes	13 (0.9)	20 (0.5)	
**BMI**			** < 0**.**001**
Underweight	152 (10.2)	668 (15.3)	
Normal	664 (44.5)	2609 (59.8)	
Overweight	262 (17.5)	592 (13.6)	
Obese	415 (27.8)	492 (11.3)	
**Systolic blood pressure**			** < 0**.**001**
Normal	707 (47.4)	2392 (54.8)	
High	786 (52.6)	1969 (45.2)	
**Diastolic blood pressure**			0.06
Normal	1302 (87.2)	3883 (89)	
High	191 (12.8)	478 (11)	
**1- Laboratory test**			
**Calcium**			** < 0**.**001**
Normal	1359 (91)	4093 (93.9)	
High	119 (8)	223 (5.1)	
Low	15 (1)	45 (1)	
**RBC**			0.61
Normal	1301 (87.1)	3842 (88.1)	
High	172 (11.5)	467 (10.7)	
Low	20 (1.3)	52 (1.2)	
**Hemoglobin**			**0**.**03**
Normal	1301 (87.1)	3677 (84.3)	
High	8 (0.5)	29 (0.7)	
Low	184 (12.3)	655 (15)	
**Ferritin**			** < 0**.**001**
Normal	1297 (86.9)	3709 (85)	
High	8 (0.5)	3 (0.1)	
Low	188 (12.6)	649 (14.9)	
**Alkaline phosphatase**			0.65
Normal	1187 (79.5)	3426 (78.6)	
High	292 (19.6)	885 (20.3)	
Low	14 (0.9)	50 (1.1)	
**Phosphate**			0.28
Normal	1091 (73.1)	3094 (70.9)	
High	393 (26.3)	1241 (28.5)	
Low	9 (0.6)	26 (0.6)	
**2- Dietary behavior**			
**Eating breakfast**			0.08
No	365 (24.4)	1167 (26.8)	
Yes	1128 (75.6)	3194 (73.2)	
**Main meals per day**			0.12
No main meal	37 (2.5)	155 (3.6)	
1–2	606 (40.6)	1783 (40.9)	
≥ 3	850 (56.9)	2423 (55.6)	
**Snacks per day**			** < 0**.**001**
No snack	160 (10.7)	329 (7.5)	
1–2	1005 (67.3)	2968 (68.1)	
≥ 3	328 (22)	1064 (24.4)	
**Fruits per day**			0.25
Not daily	936 (62.7)	2820 (64.7)	
1–2	392 (26.3)	1117 (25.6)	
≥ 3	165 (11.1)	424 (9.7)	
**Vegetables per day**			0.90
Not daily	686 (45.9)	2031 (46.6)	
1–2	692 (46.3)	1991 (45.7)	
≥ 3	115 (7.7)	339 (7.8)	
**Carbonated beverage per day**			**0**.**04**
No or not daily	545 (36.5)	1726 (39.6)	
≥ 1	948 (63.5)	2635 (60.4)	
**Energy drinking per day**			0.59
No or not daily	1158 (77.6)	3412 (78.2)	
≥ 1	335 (22.4)	949 (21.8)	
**Milk drinking per day**			0.93
No or not daily	830 (55.6)	2417 (55.4)	
≥ 1	663 (44.4)	1944 (44.6)	
**Number of days eating fast-food meals per week**			0.63
No fast-food meals	313 (21)	947 (21.7)	
1–2 days	706 (47.3)	2001 (45.9)	
≥ 3	474 (31.7)	1413 (32.4)	
**Diet for weight loss**			**0**.**02**
No	1120 (75)	3398 (77.9)	
Yes	373 (25)	963 (2.1)	
**3- Activities**			
**Days of exercising ≥ 30 min/last week**			0.07
< 3 days	1056 (70.7)	3192 (73.2)	
≥ 3 days	437 (29.3)	1169 (26.8)	
**Daily hours spent in watching TV**			0.29
Don’t watch or < 1 h	507 (34)	1416 (32.5)	
≥ 1 h	986 (66)	2945 (67.5)	
**Daily hours spent in playing video games**			**0**.**01**
Don’t play or < 1 h	1095 (73.3)	3356 (77)	
≥ 1 h	398 (26.7)	1005 (23)	
**4- Tobacco and substance use**			
**Smoke cigarette**			**0**.**03**
No	1198 (80.2)	3613 (82.8)	
Yes	295 (19.8)	748 (17.2)	
**Smoke sheesha**			**0**.**01**
No	1301 (87.1)	3911 (89.7)	
Yes	192 (12.9)	450 (10.3)	
**Sniffed solvent**			**0**.**02**
No	1265 (84.7)	3583 (82.2)	
Yes	228 (15.3)	778 (17.8)	
**Ever smoked marijuana/hashish**			0.40
No	1473 (98.7)	4314 (98.9)	
Yes	20 (1.3)	47 (1.1)	
**Ever had Alcohol**			0.31
No	1468 (98.3)	4304 (98.7)	
Yes	25 (1.7)	57 (1.3)	
**Ever had an illicit substance (e.g. captagon or other stimulants)**			1
No	1470 (98.5)	4294 (98.5)	
Yes	23 (1.5)	67 (1.5)	

*BMI* body mass index, *IQR* interquartile range, *min* minutes, *N* number of participants, *P P* value (t-test for continuous variables and χ^2^ for categorical variables), *RBC* red blood cells count, *TV* television.

Significant values are in bold.

### Multivariate-adjusted ORs and 95% CI for dyslipidemia

The final logistic multivariate regression model for factors associated with dyslipidemia, is presented in Table 4. In brief, the odds of dyslipidemia among males were 2.19 times as in females. BMI-for-age was significantly associated with dyslipidemia; increased BMI was associated with increased odds of dyslipidemia. Compared to participants with normal weight, odds of dyslipidemia was 20% less likely among participants with underweight, 76% more likely in those with overweight, and approximately threefold greater among obese adolescents.

**Table 4:** Univariate and multivariate logistic regression analysis model for variables associated with dyslipidemia.

Factor	Univariate analysis Odd Ratio (95% CI)	*P*	Multivariate analysis Odd Ratio (95% CI)	*P*
**Gender**		** < 0**.**001**		** < 0**.**001**
Male	2.32 (1.87–2.88)		2.19 (1.78–2.70)	
Female	Reference		Reference	
**BMI**		** < 0**.**001**		** < 0**.**001**
Underweight	0.89 (0.74–1.09)		0.80 (0.69–0.94)	
Normal	Reference		Reference	
Overweight	1.80 (1.1.55–2.10)		1.76 (1.50–2.06)	
Obese	3.23 (2.69–3.88)		2.80 (2.34–3.34)	
**Systolic blood pressure**		** < 0**.**001**		0.47
High	1.37 (1.23–1.54)		1.04 (0.93–1.17)	
Normal	Reference		Reference	
**Calcium**		** < 0**.**001**		0.07
High	1.52 (1.26–1.83)		1.31 (1.03–1.66)	
Low	1.25 (0.54–3.06)		1.52 (0.76–2.07)	
Normal	Reference		Reference	
**Ferritin**		**0**.**02**		**0**.**04**
High	8.07 (1.90–34.32)		7.02 (1.49–34.79)	
Low	0.90 (0.76–1.06)		0.82 (0.67–1.01)	
Normal	Reference		Reference	
**Snacks per day**		** < 0**.**001**		0.45
1–2	0.73 (0.54–0.97)		0.83 (0.62–1.12)	
≥ 3	0.63 (0.51–0.79)		0.86 (0.63–1.16)	
No snack	Reference		Reference	
**Carbonated beverage per day**		**0**.**01**		**0**.**03**
≥ 1	1.18 (1.05–1.32)		1.10 (1.00–1.20)	
No or not daily	Reference		Reference	
**Daily hours spent in playing video games**		**0**.**01**		0.50
≥ 1 h	1.20 (1.02–1.41)		0.95 (0.83–1.10)	
Don’t play or < 1 h	Reference		Reference	

*95% CI* 95% confidence interval, *BMI* body mass index, *P P* value (Wald test).

Significant values are in bold.

For laboratory test results, adolescents with high serum ferritin had 7.02 times the odds of having dyslipidemia. Inversely, those who had a low level of ferritin were 18% less likely to develop dyslipidemia than those with normal ferritin values. Further, elevated serum calcium was associated increased odds of dyslipidemia, but this association did not attain statistical significance (*p* = 0.07).

In regard to dietary behaviors assessed in this study, participants reporting ≥ 1 daily intake of carbonated beverage were 10% more likely to develop dyslipidemia than those reporting no or not daily intake. However, other dietary behaviors, and age, chronic illness, diabetes, high blood pressure, laboratory tests (RBC, hemoglobin, alkaline phosphatase, and phosphate), lifestyle factors, physical activity, and tobacco and substance use did not show statistically significant association with dyslipidemia.

## Discussion

This is the first study to estimate the prevalence of dyslipidemia among adolescents across all the 13 regions of Saudi Arabia. Our results demonstrate that one in every four adolescents in Saudi Arabia has dyslipidemia.

In comparison to international studies, the overall prevalence of dyslipidemia among Saudi adolescents is higher than Korean (19.7%) and Spanish (19.2%) and slightly lower than American (27%) and Brazilian (29%) adolescents^22–25^. The comparison with previous Saudi studies cannot be made because of their limitations; those studies included participants with different ages, followed the adult treatment panel (ATP III) guidelines for dyslipidemia definition or were performed in one city^15–17^. It is difficult to explain the differences in the prevalence of dyslipidemia in Saudi regions. Although it has not been investigated, it may be due to differences in genetic predisposition, lifestyle factors, and dietary habits. This requires further investigation in the future.

In general, young females tend to have higher lipid values than young males, but higher TG and similar estimates for Non-HDL-C; these results parallel to our findings^26^. Males had higher TG means and lower HDL-C, while females had higher means of TC and LDL-C, and no statistically significant differences in Non-HDL-C. When comparing our findings with the previous reports in Saudi Arabia, some differences are noted. For example, El-Hazami and Warsi showed a lower mean in TC (3.9) and TG (0.80)^16^. While Al-Shehri et al.^15^ showed similar findings to our result with mean levels of TC, LDL-C being higher in girls, however, they found that mean TG was higher among girls. Interestingly, their overall means of lipid parameters were higher than ours regardless of age or sex.

Similar findings to our study were observed in 13,579 European non-obese children aged 2.0–10.9 years; boys had lower mean TC (3.981 vs. 4.087 mmol/L) and LDL-C (2.297 vs. 2.435 mmol/L) than girls. However, TG was lower in boys (0.509 vs. 0.542 mmol/L), and HDL-C was higher (1.414 vs. 1.368 mmol/L)^27^. Triglyceride level is strongly linked to obesity and increases with age^28^; thus, it is not surprising that the mean TG in the former study is lower than our finding.

In terms of sex differences, our study is also in line with the findings from the 2011–2014 National Health and Nutrition Examination Survey (NHANES) from the USA, which estimates the prevalence of high TC is lower in boys (5.9%) than in girls (8.9%), and boys had a higher prevalence of low HDL-C (14.8%) than in girls (12.0%). However, the NHANES study showed sex differences in the prevalence of higher non-HDL-C in girls (9.4%) compared to boys (7.5%), which is not the case in our study^24^. The potential reason for the similarity of our finding to the USA is that most of the Saudi youth population have adopted the Western, mainly American, lifestyle with the typical high-fat dense diet and sedentary habits. The gender variation in dyslipidemia prevalence is quite interesting; it has been shown that the prevalence of dyslipidemia is higher in young males than in females, and the mechanism behind this difference is largely unknown, which needs further studies. However, some studies attributed this difference to the impact of estrogen on lipid metabolism^24,29^.

Comparable studies from the following countries revealed abnormal values; in Ghana (12.1% for TC, 4.5% for TG, 28.4% for HDL-C, and 9.2% for LDL-C), in Brazil (11.2% for TC, 15.8% for HDL-C, 10.8% for LDL-C and 4.7% for TG), and in Korea (high LDL-C, high TG, and low HDL-C was 6.5%, 4.7%, 10.1%, and 7.1%), respectively^22,25,30^. The differences between countries and studies in the overall prevalence of dyslipidemia, sex differences, and percentage of abnormal lipid levels could be attributed to variations in methodology (age group, lipid cutoff reference, seasonality or fasting state), lifestyle factors, such as dietary habits, demographic features, genetic background and time interval between studies.

As per NHANES III and previous studies from different countries, it has been documented that lipid levels, particularly TC and LDL-C increase during puberty then decrease in late adolescence^26,27,31^. However, this was not observed in this study.

Regarding factors associated with dyslipidemia, we found that overweight and obese adolescents have a higher rate of dyslipidemia. This finding is in line with previous report from NHANES III, specifically high TC, low HDL-C, high TG, and high Non-HDL-C^24,30,32^. Further, the prevalence of overweight and obesity among children and adolescents has increased worldwide and in Saudi Arabia; thus, the rate of dyslipidemia is expected to trend up^13,33^. Furthermore, American Heart Association (AHA), demonstrated that being overweight and obese is associated with having higher bad cholesterol and lower good cholesterol. Being on a program for weight loss would help improve lipid figures^28^. Moreover, this result may suggest that this prevalence is mainly due to secondary dyslipidemia, which is associated with higher BMI^2^. In addition, a previous publication of the Jeeluna study showed and discussed further the importance of physical activities interventions to this population^14^. Therefore, this result supports the need and the importance of awareness programs and interventions, such as physical activity and dietary change, to reduce weight and decrease dyslipidemia prevalence.

The biochemical findings suggest that serum ferritin is related to dyslipidemia. However, the 95% confidence interval for serum ferritin range was wide, probably owing to the low number of participants with high ferritin. High serum ferritin was associated with increased odds of dyslipidemia, and low serum ferritin was associated with decreased odds of being dyslipidemic. This result is consistent with Korean adolescents’ results; dyslipidemia was statistically significantly associated with serum ferritin, particularly in boys more than in girls^34^.

Besides genetic factors that affect lipoprotein levels, secondary factors such as lifestyle, behavioral and eating habits may impact lipid levels, including diet and physical activity. This study show that daily intake of carbonated beverage is associated with 10% increase in the likelihood of dyslipidemia. A prospective cohort study conducted in USA found that daily intake of sugar-sweetened beverages was associated with adverse changes in high-density lipoprotein cholesterol and triglyceride concentrations and a high risk of incident dyslipidemia^35^. Other dietary behaviors, lifestyle factors, physical activity, and tobacco and substance use did not show statistically significant association with dyslipidemia. Other studies have shown a conflicting association in the adolescents’ age group which could be related to the methodology of studies being self-reported data and the difference in geographic and genetic background^30,36,37^.

## Conclusion

In conclusion, this study's finding suggests that at least one out of every four adolescents in Saudi Arabia is dyslipidemic. The low HDL-C level was the most prevalent type of dyslipidemia among Saudi adolescents, followed by triglycerides. Females were statistically significantly had a higher prevalence of TC, while males had lower HDL-C and higher triglycerides. After controlling for all significant covariates, a higher prevalence of dyslipidemia was observed in males, overweight/obese, and those with an elevated serum calcium concentration and serum ferritin.

Dyslipidemia begins in adolescence and may proceed to adulthood. Hence, it is critical to detect dyslipidemia at an early stage of life, as early intervention may reduce its associated morbidity and mortality due to atherosclerosis and CVD in adulthood. This study’s results suggested that policymakers should urgently develop public health interventions, such as starting early health education and public health programs aiming to improve physical activities and diet habits to manage and control lipid abnormalities among Saudi Arabian adolescents. Further studies are needed to assess the regional variations in lipid measurement and the relationship between diet and dyslipidemia.

## Strengths and limitations

One of this study’s strengths is the very large sample size representing both males’ and females’ adolescents from all 13 regions of Saudi Arabia. Moreover, this is the first study to draw a comprehensive picture of the lipid profile panel, sex differences, and the factors associated with dyslipidemia among Saudi adolescents aged 10–19 years old. However, some limitations of this study should be acknowledged. First, because of the nature of the cross-sectional type of study and measurements taken in a single shot, causality cannot be established. Second, self-reported data may be prone to recall bias.

## Data Availability

We accessed Jeeluna Survey Study data under special license and agreement with KAIMRC. Currently data are not publicly available but could be made available from the authors upon reasonable request, with the permission of KAIMRC. Requests should be sent to the corresponding author Prof. Motasim Badri (badrim@ksau-hs.edu.sa).
